# Maternal and neonatal outcomes in transverse and vertical skin incision for placenta previa: Skin incision for placenta previa

**DOI:** 10.1186/s12884-021-03923-1

**Published:** 2021-06-24

**Authors:** Dazhi Fan, Huishan Zhang, Jiaming Rao, Dongxin Lin, Shuzhen Wu, Pengsheng Li, Gengdong Chen, Zixing Zhou, Juan Liu, Ting Chen, Fengying Chen, Xiaoling Guo, Zhengping Liu

**Affiliations:** 1 Foshan Institute of Fetal Medicine, Affiliated Foshan Maternity & Child Healthcare Hospital, Southern Medical University, Foshan, 528000 Guangdong China; 2 Department of Obstetrics, Affiliated Foshan Maternity & Child Healthcare Hospital, Southern Medical University, Foshan, 528000 Guangdong China; 3 Department of Foetal Ultrasonic, Affiliated Foshan Maternity & Child Healthcare Hospital, Southern Medical University, Foshan, 528000 Guangdong China; 4 Department of Radiology, Affiliated Foshan Maternity & Child Healthcare Hospital, Southern Medical University, Foshan, 528000 Guangdong China

**Keywords:** Placenta previa, Cesarean delivery, Skin incision, Maternal and neonatal morbidity

## Abstract

**Background:**

Placenta previa, a serious obstetric issue, should be managed by experienced teams. The safe and appropriate mode of delivery for placenta previa is by cesarean delivery. However, no studies were found comparing either maternal or neonatal outcomes for different skin incision in women with placenta previa. The aim of this study was to compare maternal and neonatal outcomes by skin incision types (transverse compared with vertical) in a large cohort of women with placenta previa who were undergoing cesarean delivery.

**Methods:**

This was a retrospective cohort study carried out between January 2014 and June 2019. All pregnant women with placenta previa had confirmed by ultrasonologist before delivery and obstetrician at delivery. The primary outcome was the estimated blood loss during the surgery and within the first 24 hours postoperatively. Mean (standard deviation), median (interquartile range) or frequency (percentage) was reported to variables. Appropriate parametric and nonparametric tests were used to analyses.

**Results:**

The study included 1098 complete records, 332 (30.24%) cases in the vertical skin incision group and 766 (69.76%) cases in the transverse skin incision group. Those with vertical incision showed a higher percentage of preterm delivery, anterior placenta, abnormally invasive placenta, and history of previous cesarean delivery, and a lower percentage of first pregnancy, in vitro fertilization, and emergency cesarean delivery. After controlling for confounding factors, higher incidence of post-partum hemorrhage (OR 5.47, 95% CI 3.84–7.79), maternal intensive care unit (OR 4.30, 95% CI 2.86–6.45), transfusion (OR 5.97, 95% CI 4.15–8.58), and 5-min APGAR< 7 (OR 9.03, 95% CI 1.83–44.49), a more estimated blood loss (β 601.85, 95%CI 458.78–744.91), and a longer length of hospital stay after delivery (β 0.54, 95%CI 0.23–0.86) were found in the vertical skin incision group.

**Conclusions:**

Our data demonstrated that transverse skin incision group showed the better perinatal outcomes in women with placenta previa. Future collaborative studies are needed to be done by centers for placenta previa to have a better understanding of the characteristics and the outcomes of the disease in the choosing skin incision.

## Background

Placenta previa, a serious obstetric issue, is defined as the placenta overlying the endocervical os [1]. It is associated with adverse maternal and fetal-neonatal outcomes including perinatal hemorrhage, hysterectomy, postpartum transfusion, septicemia, thrombophlebitis, prematurity, intrauterine growth restriction, neonatal anemia, and even maternal and fetal death [2–4]. Concomitantly with the rising incidence of cesarean delivery, use of fertility treatments, increasing maternal age, and inappropriate habits, such as smoking and cocaine use, the rate of placenta previa is also rising [5–8].

The safe and appropriate mode of delivery for placenta previa is by cesarean delivery. Various abdominal incisions have used for cesarean delivery, and these mainly include vertical and transverse skin incisions [9]. For complicated pregnant women, such as multiple prior cesarean deliveries, twins, and placenta previa, many operators advise routine use of a vertical skin incision to facilitate access to the fundus and pelvic walls [10–13]. However, several studies have compared maternal and neonatal outcomes according to the type of skin incisions, and the researchers were unable to find any explicit benefit from the use of a vertical skin incision [10, 11]. On the contrary, one study even reported that vertical skin incision was associated with a higher risk of bladder and bowel injury [14].

No studies found comparing either maternal or neonatal outcomes for different skin incisions in women with placenta previa. Thus, it is necessary to evaluate it, and this study aimed to compare maternal and neonatal outcomes by skin incision types (transverse compared with vertical) in a large cohort of women with placenta previa who were undergoing cesarean delivery.

## Methods

An institutional review board-approved (FSFY-MEC-2019-044) retrospective cohort study performed at our hospital. Medical records were reviewed retrospectively by experienced senior obstetricians and demographics, clinical characteristics, and outcomes recorded. Demographic, clinical, and outcomes data collected included age, body mass index, gestational age at delivery, gravidity, parity, history of cesarean delivery, placenta location, operation time, neonatal weight, sex of newborn, estimated blood loss during the surgery and within the first 24 hours postoperatively, transfusion, surgical injury, and length of hospital stay after delivery.

Inclusion criteria were all pregnant patients with placenta previa from January 2014 through June 2019. All cases had confirmed by ultrasonologist before delivery and obstetrician at delivery. Exclusion criteria included multiple gestations, stillbirths, and incorrect dating, and incomplete delivery records in which outcomes could not be obtained were also excluded. Finally, 1098 pregnant women were included and 169 pregnant women were excluded.

The primary outcome was the estimated blood loss during the surgery and within the first 24 hours postoperatively. Intraoperatively, blood loss was collection and measurement for a drape with a blood collection system around the abdominal wound from the abdominal cavity. Gauzes were used to collect blood from the vagina. All gauzes with blood were collected, weighed and an equivalent volume was calculated. The volume of blood loss is equal to the weight of blood loss ÷ 1.05. This stage was mainly accomplished by trained operating room nurses. Any post-cesarean delivery blood loss was also quantified. In this stage, a gynecological drape with pouch was also used to collect blood from the vagina. This stage was mainly accomplished by trained obstetric nurses, and the nurses checked in every two hours. Other outcomes recorded were post-partum hemorrhage (>1000 ml), transfusion, the number of red blood cell units and fresh frozen plasma transfused, maternal intensive care unit, hysterectomy, surgical injury, 5-min APGAR < 7, and the length of hospital stay after the delivery.

All of the transverse incisions were Pfannenstiel, and vertical incisions were midline in our institution. The decision to perform a transverse or vertical skin incision was left up to the judgment of the individual obstetrician. Random placenta margin incision was selected for the uterine incision [15]. For challenging cases (prior surgery, invasion trophoblast, ect.), various surgical techniques, such as double incision for safe extraction of the fetus, sandwich excision, metroplasty, suturing of a bladder defect, and B_Lych, were involved during cesarean delivery to control intraoperative and total blood loss. The surgical techniques were selected according to the patient’s actual condition. Meanwhile, oxytocin and/or carbetocin were also used after delivery of the placenta to reducing the postpartum hemorrhage. If necessary, intraoperative transfusion, balloon tamponade, hypogastric artery ligation, hysterectomy, postoperative transfusion, and ICU admission were performed according to standard protocols.

## Statistical Analysis

All continuous variables tested for normality using descriptive statistics for skewness and kurtosis and the Kolmogorov-Smirnov test. Descriptive statistics (mean ± standard deviation for normality continuous variables, the median and interquartile range for abnormality continuous variables, and the frequencies and percentages for categorical variables) were calculated for those with vertical versus transverse skin incision. Baseline variables and maternal delivery characteristics were compared by skin-incision type. These two groups were compared using the two-sample t-test or Mann-Whitney U test for normality or abnormality continuous data and the chi-square test or Fisher’s exact test, as deemed appropriate, for categorical variables. Logistic- or linear-regression was used to determine the association between vertical skin incision and primary and other outcomes. A multivariable regression model was developed to adjust for potential confounding factors. A result was considered statistically significant at the value of *P* < 0.05 level of significance. All analyses were performed using SPSS software (version 21.0; SPSS Inc, Chicago, IL).

This is an observational study (retrospective cohort study), and multivariable regression was used to determine the association between skin incision types and outcomes. Twenty-nine variables were included in this study. Observational studies were conducted on each variable in 15-30 sample sizes [16, 17]. A total of 870 samples were required for this study. Considering some variables have missing values, 1098 samples were enough for this study.

## Results

The study included 1098 complete records, 332 (30.24%) cases in the vertical skin incision group and 766 (69.76%) cases in the transverse skin incision group, for review during the time frame of January 2014 to June 2019. The baseline characteristics between the two comparison groups were demonstrated in Table 1 with *P* values < 0.05 considered significant.

**Table 1:** Characteristics of the study participants in the two study groups

Variables	Total (*n* = 1098)	Vertical incision (*n* = 332)	Transverse incision (*n* = 766)	t/Z/χ^2^	*P* value
Age, years, mean±sd	32.56±5.34	32.94±5.54	32.40±5.25	−1.547	0.122
BMI, kg/m^2^, mean±sd	26.40±7.37	26.19±3.27	26.49±8.57	0.587	0.557
EGA at delivery, week, mean±sd	36.36±2.41	35.97±2.00	36.53±2.55	3.886	0.001
Preterm birth (EGA < 37 weeks), n, %	599 (54.6)	233 (70.2)	366 (47.8)	46.875	0.001
Gravidity, median [IQR]	3.00 (2.00–4.00)	3.00 (2.00–4.00)	2.00 (2.00–3.00)	−5.437	0.001
First pregnancy, n, %	202 (18.4)	32 (9.6)	170 (22.2)	24.318	0.001
In vitro fertilization, n, %	141 (12.8)	27 (8.1)	114 (14.9)	9.429	0.002
Previous CD, n, %	489 (44.5)	215 (64.8)	274 (35.8)	78.796	0.001
Number of previous CDs, median [IQR]	1.00 (1.00–2.00)	1.00 (1.00–2.00)	1.00 (1.00–1.00)	−10.190	0.001
Antepartum hemorrhage, n, %	467 (42.5)	150 (45.2)	317 (41.4)	1.366	0.259
Anterior placenta	386 (35.2)	195 (58.7)	191 (24.9)	116.076	0.001
Depth of invasion, n, %					
Accreta	101 (9.2)	55 (16.6)	46 (6.0)	199.900	0.001
Increta	148 (13.5)	104 (31.3)	44 (5.7)		
Percreta	9 (0.8)	8 (2.4)	1 (0.1)		
Emergency CD, n, %	338 (30.8)	76 (22.9)	262 (34.2)	16.617	0.001
Operation time, minute, median [IQR]	55.00 (42.00–80.00)	85.00 (57.25–150.00)	47.50 (40.00–61.25)	−15.060	0.001
Incision-to-delivery intervals, minute, median [IQR]	6.00 (4.00–12.00)	12.00 (6.00–37.00)	5.00 (4.00–8.00)	−13.879	0.001
Neonatal weight, gram, median [IQR]	2745.00 (2400.00–3062.50)	2660.00 (2370.00–2920.00)	2790.00 (2420.00–3120.00)	−3.607	0.001
Male newborn, n, %	643 (58.6)	194 (58.4)	449 (58.6)	0.003	0.999

*BMI* body mass index, *CD* cesarean delivery, *EGA* estimated gestational age, *IQR* interquartile range

Those with vertical incision showed a higher percentage of preterm delivery (70.2% vs. 47.8%), anterior placenta (58.7% vs. 24.9%), abnormally invasive placenta (50.3% vs. 11.8%), and history of previous cesarean delivery (64.8% vs. 35.8%), and a lower percentage of first pregnancy (9.6% vs. 22.2%), in vitro fertilization (8.1% vs. 14.9%), and emergency cesarean delivery (22.9% vs. 34.2%). Operation time and incision-to-delivery time were longer in the vertical skin incision group (85.00 [57.25–150.00] vs. 47.50 [40.00–61.25] minutes and 12.00 [6.00–37.00] vs. 5.00 [4.00–8.00] minutes, respectively), and neonatal weight was lighter in the vertical skin incision group (2660.00 [2370.00–2920.00] vs. 2790.00 [2420.00–3120.00] grams).

As shown in Table 2, the primary outcome was significantly higher in the vertical skin incision cases when compared with the transverse skin incision cases (estimated blood loss: β 1420.66, 95%CI 1266.68–1574.63). Vertical incision was also associated with a higher incidence of post-partum hemorrhage (68.7% vs. 16.3%, OR 11.24, 95% CI 8.32–15.19), maternal intensive care unit (48.5% vs. 8.5%, OR 10.15, 95% CI 7.28–14.17), transfusion (81.6% vs. 27.8%, OR 11.53, 95% CI 8.38–15.88), subtotal hysterectomy (2.7% vs. 0.4%, OR 7.09, 95% CI 1.91–26.35), bladder injury (1.2% vs. 0.1%, OR 9.33, 95% CI 1.04–83.79), and 5-min APGAR< 7 (3.0% vs. 0.4%, OR 7.90, 95% CI 2.16–28.89). The length of hospital stay after the delivery was longer in the vertical skin incision group (β 1.29, 95%CI 1.00–1.57).

**Table 2:** Maternal and neonatal outcomes in the two study groups

Variables	Total (*n* = 1098)	Vertical incision (*n* = 332)	Transverse incision (*n* = 766)	OR (95%CI) */β (95%CI)*	t/Z/χ^2^	*P* value
EBL, ml, median [IQR]	600.00 (400.00–1200.00)	1500.00 (800.00–3000.00)	450.00 (350.00–700.00)	1420.66 (1266.68–1574.63)	−17.661	0.001
PPH, n, %	353 (32.1)	228 (68.7)	125 (16.3)	11.24 (8.32–15.19)	291.054	0.001
Maternal ICU, n, %	226 (20.6)	161 (48.5)	65 (8.5)	10.15 (7.28–14.17)	226.801	0.001
Transfusion, n, %	484 (44.1)	271 (81.6)	213 (27.8)	11.53 (8.38–15.88)	272.169	0.001
RBC units transfused, median [IQR]	0.00 (0.00–4.00)	6.00 (2.00–10.00)	0.00 (0.00–2.00)	6.77 (5.72–7.82)	−18.094	0.001
Plasma ml transfused, median [IQR]	0.00 (0.00–0.00)	400.00 (0.00–800.00)	0.00 (0.00–0.00)	389.04 (343.18–434.90)	−16.655	0.001
Subtotal hysterectomy, n, %	12 (1.1)	9 (2.7)	3 (0.4)	7.09 (1.91–26.35)	11.525	0.002
Surgical injury	46 (4.2)	12 (3.6)	34 (4.4)	0.81 (0.41–1.58)	0.392	0.624
Bladder Injury	5 (0.5)	4 (1.2)	1 (0.1)	9.33 (1.04–83.79)	5.897	0.031
Blood Vessels Injury	41 (3.7)	8 (2.4)	33 (4.3)	0.55 (0.25–1.20)	2.322	0.165
Postoperative hospital stay, days, median [IQR]	4.00 (3.00–5.00)	5.00 (4.00–6.00)	4.00 (3.00–4.00)	1.29 (1.00–1.57)	−11.141	0.001
5-min APGAR < 7, n, %	13 (1.2)	10 (3.0)	3 (0.4)	7.90 (2.16–28.89)	13.594	0.001

*EBL* estimated blood loss, *ICU* intensive care unit, *IQR* interquartile range, *PPH* post-partum hemorrhage, *RBC* red blood cell

After controlling for baseline differences between the two groups (estimated gestational age at delivery, gravidity, in vitro fertilization, number of previous cesarean delivery, anterior placenta, depth of invasion, and neonatal weight), there was also a higher incidence of post-partum hemorrhage (OR 5.47, 95% CI 3.84–7.79), maternal intensive care unit (OR 4.30, 95% CI 2.86–6.45), transfusion (OR 5.97, 95% CI 4.15–8.58), and 5-min APGAR< 7 (OR 9.03, 95% CI 1.83–44.49), a more estimated blood loss (β 601.85, 95%CI 458.78–744.91), and a longer length of hospital stay after delivery (β 0.54, 95%CI 0.23–0.86) in the vertical skin incision group (Table 3).

**Table 3:** Results of maternal and neonatal outcomes after adjusting for EGA at delivery, gravidity, in vitro fertilization, number of previous CD, anterior placenta, depth of invasion, and neonatal weight.

Variables	*OR (95%CI)/β (95%CI)*	*P* value
EBL	601.85 (458.78–744.91)	0.001
PPH	5.47 (3.84–7.79)	0.001
Transfusion	5.97 (4.15–8.58)	0.001
Maternal ICU	4.30 (2.86–6.45)	0.001
Subtotal hysterectomy	0.69 (0.14–3.42)	0.651
Bladder Injury	0.75 (0.06–9.45)	0.823
Length of hospital stay	0.54 (0.23–0.86)	0.001
5-min APGAR < 7	9.03 (1.83–44.49)	0.007

*CD* cesarean delivery, *EBL* estimated blood loss, *EGA* estimated gestational age, *ICU* intensive care unit, *PPH* post-partum hemorrhage

## Discussion

This analysis of a large cohort of placenta previa women undergoing cesarean delivery sought to answer the question of whether the skin incision, vertical compared with transverse, is associated with a difference in the maternal and neonatal outcomes. In this study, we found that transverse skin incision group showed the better perinatal outcomes, including less blood loss, lower post-partum hemorrhage, transfusion, maternal ICU, and 5-min APGAR < 7, and a shorter length of hospital stay after the delivery for women with placenta previa.

It is difficult to compare our results with the literature because previous anecdotal evidence assessing the type of incision in placenta previa pregnancies came only from the experience of the surgeons or other plausible empirical extrapolations. To the best of our knowledge, this is the first study comparing transverse versus vertical skin incision in women with placenta previa having a cesarean delivery. Our results will provide some evidence for this troublesome clinical problem and fill this research gap.

The vertical skin incision considered to provide faster access to the abdominal cavity [18]. It is regarded by some obstetricians as a method to decrease incision-to-delivery time when performing a cesarean delivery in women with a complicated pregnancy [19, 20]. However, our results did not support it. Inversely, a longer incision-to-delivery time was found in the vertical incision group. The differences in outcome may be not truly related to the choice of incision because of inherently greater risk in the vertical incision group. The placenta in the vertical incision group occupied a greater proportion of the anterior uterine wall and abnormally invasive placenta than in the transverse group in this study. The longer operative time suggested the vertical group had an intrinsically greater risk and more likely reflects underlying complications.

Cesarean delivery is a complicated procedure, and there is a risk of inadvertent injury such as bladder, bowel, and blood vessel injury [21]. In this study, we found vertical skin incision could increase the risk of bladder injury, which is consistent with the previous research. Makoha et al. also reported that vertical skin incision was associated with higher bladder and bowel injury [14]. However, the difference disappeared after adjusting for confounding factors in our study.

Post wound infection is one of the most common postoperative complications after the cesarean section [22, 23]. A recent report from Ethiopia showed that vertical skin incision was associated with higher rates of surgical site infection following the cesarean section [24]. In our population, none of the patients found postoperative wound infection. This might be because standardized surgical procedures and prophylactic antibiotics performed during the perioperative period of a pregnancy complicated by placenta previa. Standardized protocols for intraoperative transfusion, balloon tamponade, hypogastric artery ligation, hysterectomy, incision choice, postoperative transfusion, ICU admission, etc were need to be developed during cesarean delivery for complicated pregnant women, such as placenta previa. Standard protocols rather than left up to the judgment of the individual surgeon or obstetrician should be provided during cesarean delivery. Without standardization, there is substantial risk of bias, even with relatively large sample size.

What needs to emphasize is that there is more severe pregnant, including more gravidity, earlier gestational age, antepartum hemorrhage, previous cesarean delivery, anterior placenta, and abnormally invasive placenta in the vertical incision. These factors can increase the incidence of adverse postpartum outcomes. However, after controlling for confounders, transverse skin incision group still showed the better perinatal outcomes. It is not clear why the transverse incision type is associated with better perinatal outcomes. This may be because surgeons have to consciously enlarge the vertical skin incision, including width and length, to cover the previous incision due to previous scarring, thereby making trauma more likely. In addition, it is possible that surgeons place too much confidence in the safety of a vertical incision and therefore act with less caution than would be exercised with transverse incision, because vertical skin incision is regarded by some obstetricians as a method to decrease the rate of complications when performing a cesarean delivery in women with severe complications.

The strengths of this study include the large and adequate sample size, and that our findings are relatively new adding knowledge to the literature given the existing scarce data regarding the clinical usefulness of skin incision in placenta previa pregnant. All of the subjects included in this study have confirmed by obstetricians at delivery, and thus, selection bias dependent on inaccurate antenatal ultrasound diagnosis has been avoided. Patients were managed in a single institution, and therefore, the observed differences in outcome can be reliably related to differences in management at the time of delivery rather than substantive differences in human or facility resources.

Our results must be interpreted with caution because our study was limited by its retrospective observational nature. The study attempted to compensate for various risk factors by doing a regression analysis. We were able to isolate the effects of several, but not all variables that might have influenced the outcome. Some variables, such as use of balloon tamponade, selective artery ligation (ovarian, uterine, hypogastric), and uterine incision type, might have been useful to assess the outcomes, but they were not collected in this study. However, its feasibility of a randomized control trial seems challenging to perform in these pregnant women. For challenging cases, various surgical techniques can be applied to the uterus, and surgeons may have been more likely to choose a vertical skin incision. Complications such as hemorrhage would then be more likely attributable to the choice of uterine incision, density of adhesions, etc. than to the nature of the skin incision. The decision regarding which type of skin incision used should be made by the operating team and to individualize the incision in each case. The location of the placenta, abnormally invasive placenta, the likelihood of intraoperative complications, maternal body habitus, gestational age, and preference of the operating obstetrician should all taken into consideration. In addition, we should notice that differences in surgical experience and skills were difficult to control in any study comparing surgical methods. Also, it is often quite difficult from chart review to judge the difficulty of handling any complications that occurred. Besides, we do not have assessed long-term morbidity and mortality among mothers and infants. More extensive trials, which include long-term pain, development of hernia, appearance of and satisfaction with scar, and outcomes in a subsequent pregnancy, would be required to assess these issues.

## Conclusions

Our data demonstrated that transverse skin incision group showed the better perinatal outcomes in women with placenta previa. What needs to be emphasized was that these results were not as the factors for decision-making for how to perform skin incision because major findings from this study were not resulted from the choice of skin-incision, but from the patients’ background including higher-risk of placenta previa that is necessary to perform operation with vertical skin incision. Future collaborative studies are needed to be done by centers for placenta previa to have a better understanding of the characteristics and the outcomes of the disease in choosing skin incision.

## Data Availability

The datasets used and/or analysed during the current study are available from the corresponding author on reasonable request.

## Abbreviations

CI: confidence interval
ICU: intensive care unit
OR: odds ratio
